# Brain age predicts mortality

**DOI:** 10.1038/mp.2017.62

**Published:** 2017-04-25

**Authors:** J H Cole, S J Ritchie, M E Bastin, M C Valdés Hernández, S Muñoz Maniega, N Royle, J Corley, A Pattie, S E Harris, Q Zhang, N R Wray, P Redmond, R E Marioni, J M Starr, S R Cox, J M Wardlaw, D J Sharp, I J Deary

**Affiliations:** 1 Computational, Cognitive and Clinical Neuroimaging Laboratory, Department of Medicine, Imperial College London, London, UK; 2 Centre for Cognitive Ageing and Cognitive Epidemiology, University of Edinburgh, Edinburgh, UK; 3 Department of Psychology, University of Edinburgh, Edinburgh, UK; 4 Brain Research Imaging Centre, Centre for Clinical Brain Sciences, University of Edinburgh, Edinburgh, UK; 5 Centre for Genomic and Experimental Medicine, MRC Institute of Genetics & Molecular Medicine, University of Edinburgh, Edinburgh, UK; 6 Institute for Molecular Bioscience, The University of Queensland, QLD, Australia; 7 Queensland Brain Institute, The University of Queensland, QLD, Australia

## Abstract

Age-associated disease and disability are placing a growing burden on society. However, ageing does not affect people uniformly. Hence, markers of the underlying biological ageing process are needed to help identify people at increased risk of age-associated physical and cognitive impairments and ultimately, death. Here, we present such a biomarker, ‘brain-predicted age’, derived using structural neuroimaging. Brain-predicted age was calculated using machine-learning analysis, trained on neuroimaging data from a large healthy reference sample (*N*=2001), then tested in the Lothian Birth Cohort 1936 (*N*=669), to determine relationships with age-associated functional measures and mortality. Having a brain-predicted age indicative of an older-appearing brain was associated with: weaker grip strength, poorer lung function, slower walking speed, lower fluid intelligence, higher allostatic load and increased mortality risk. Furthermore, while combining brain-predicted age with grey matter and cerebrospinal fluid volumes (themselves strong predictors) not did improve mortality risk prediction, the combination of brain-predicted age and DNA-methylation-predicted age did. This indicates that neuroimaging and epigenetics measures of ageing can provide complementary data regarding health outcomes. Our study introduces a clinically-relevant neuroimaging ageing biomarker and demonstrates that combining distinct measurements of biological ageing further helps to determine risk of age-related deterioration and death.

## Introduction

As the global population ages, the burden of disease is increasing.^1^ This has motivated research to understand the biological links between ageing and disease risk. There is substantial heterogeneity in how the ageing process affects different individuals, indicating that people age at different rates, due to both genetic and environmental influences. If the biological characteristics of these different rates of ageing can be measured, then biomarkers of individual differences in the ageing process might help improve predictions of mortality and morbidity. Such biomarkers could potentially identify those at risk of age-associated health problems years before symptoms appear, and be used as outcome measures in trials of therapeutics aimed at delaying the onset of age-related disease. Many different ageing biomarkers have been proposed, which tap into different cellular and molecular aspects of ageing. For example, the so-called ‘epigenetic clock’^2, 3^ uses measurements of DNA-methylation status at CpG sites across the genome, which can be converted into an age prediction which correlates highly with chronological age in healthy individuals. Other candidate ageing biomarkers include leucocyte telomere length,^4^ N-glycan profile^5^ and Ink4a/Arf locus expression.^6^ This diverse list of candidate ageing biomarkers reflects the involvement of multiple biological systems and the overall complexity of the ageing process in humans.^7^

Neurological aspects of ageing, such as cognitive decline and dementia, are particularly deleterious to general health and well-being.^8^ Brain structure is well-known to alter throughout life,^9^ and deviations from this typical brain ageing trajectory, in terms of increased brain atrophy for a given age, may well reflect latent neuropathological influences. A reliable and valid brain-based biomarker of ageing, that identifies individuals deviating from a healthy brain ageing trajectory, could have great utility in efforts to combat age-associated neurodegeneration and its consequences.

Neuroimaging is a powerful tool for deriving *in vivo* data on the ageing brain, demonstrating both global and spatially-localised relationships with normal ageing,^10, 11^ and with age-associated cognitive decline.^12, 13, 14, 15^ Recently, multivariate methods have been developed to define statistical models of healthy brain ageing. Using machine-learning analysis of neuroimaging data, chronological age can be accurately predicted in healthy individuals.^16^ This provides a method of measuring whether a person’s brain appears younger or older than their chronological age. Using this model, deviations from healthy brain ageing have been identified in Alzheimer’s disease,^17^ mild cognitive impairment,^18^ schizophrenia^19^ and have been related to cognitive impairment after traumatic brain injury.^20^ Furthermore, protective factors have been associated with a positive influence on brain ageing. For example, years of education, physical exercise and practicing meditation were recently linked to having younger-appearing brains.^21, 22^

As these multivariate neuroimaging measures have been associated with age-related pathology and cognitive impairment, this raises the possibility that brain-based age predictions could be used as an ageing biomarker. A viable ageing biomarker must relate to the risk of mortality and age-associated morbidity,^23^ particularly if it is to have clinical utility. To establish what the consequences of having a brain that appears older or younger than average for one’s chronological age, we estimated ‘brain-predicted age’ in a large, narrow age-range population cohort of older adults (Lothian Birth Cohort 1936 (LBC1936), *N*=669), using structural neuroimaging (T1-weighted magnetic resonance imaging (MRI)). We tested the association between brain-predicted age difference (brain-PAD; calculated as brain-predicted age minus chronological age) and: mortality risk, disease prevalence, measures of physical and mental fitness (grip strength, walking speed, lung function and general fluid intelligence), and a composite measure of biological health (allostatic load). We hypothesised that ‘older’ brain-PAD would be associated with earlier mortality and more morbidity, poorer physical and cognitive fitness, and greater allostatic load.

Further, it has been proposed that biological ageing occurs at different rates to different tissues or cells within the same person, the so-called ‘mosaic of ageing’.^24^ Hence, complementary information could be gained by combining ageing biomarkers derived from different sources. Conversely, if ageing occurs uniformly across the body, then diverse ageing biomarkers should correlate highly. Here we explored these possibilities by examining brain-predicted age in relation to molecular-genetic ageing biomarkers. We tested brain-PAD in combination with DNA-methylation-based age predictions using the ‘epigenetic-clock’^3^ and leukocyte telomere length, both previously associated with mortality,^25, 26^ examining their influence on the relationship with age-related outcome measures. Finally, we considered how brain-PAD related to more conventional imaging measures, previously shown to relate to ageing.

## Materials and methods

Full details of the participants, data acquisition and statistical methods used in the study are included in Supplementary Methods.

### Participants—Lothian Birth Cohort 1936

The LBC1936 is a longitudinal study of ageing based in the Edinburgh and Lothians area of Scotland, UK.^27, 28^ Most of the participants had taken part in the Scottish Mental Survey of 1947, which involved a test of general cognitive ability for almost all 11-year old children in the country at that time.^29^ At the first wave, 1,091 participants attended for cognitive and medical testing (mean age 70 years, 548=male, 543=female). MRI testing began at the second wave, when 866 individuals attended for cognitive, medical testing (mean age 73 years, 448=male, 418=female), of whom 669 (352=male, 317=female) had MRI. This final cohort provided the data that were included in present analysis (Table 1). The vast majority of participants were cognitively normal according to mini-mental state examination, with 99.3% scoring ⩾24. Ethical approval for the LBC1936 was obtained from the Multi-Centre Research Ethics Committee for Scotland (MREC/01/0/56) and the Lothian Research Ethics Committee (LREC/2003/2/29). Written informed consent was obtained from all subjects.

### Participants—brain-predicted age training cohort

Further 2001 healthy individuals (age mean=36.95±18.12 years; age range=18–90 years; males=1016; females=985) comprised the brain-predicted age training cohort. These data were obtained via publicly-available repositories (Supplementary Table 5) and were screened according to local study protocols to ensure that they were free of neurological and psychiatric disorders, had no history of head trauma and other major medical conditions. Ethical approval for each initial study and subsequent data sharing was verified for each data repository.

### Brain age prediction methods

**Figure 1:**
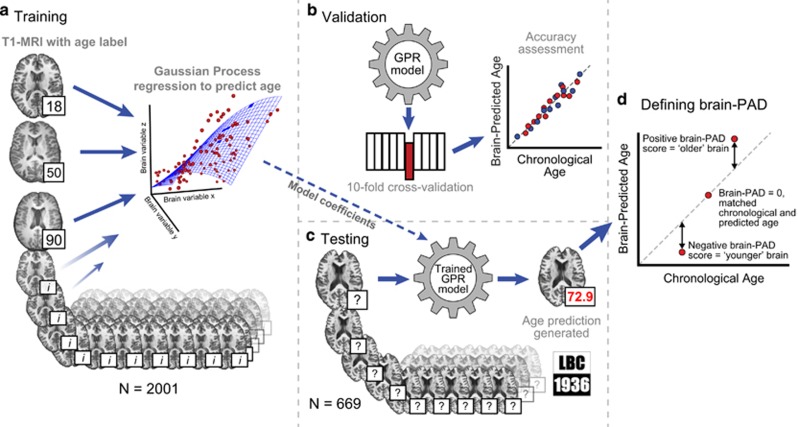
Overview of study methods. Illustration of the methods used to generate brain-predict ages. 3D T1-weighted MRI scans were segmented into grey and WM before being normalised in common space using non-linear spatial registration. Normalised grey and WM images were concatenated and converted into vectors for each subject. These vectors were then projected into an NxN similarity matrix based on vector dot-products. (**a**) Once in similarity matrix form the training subjects’ data were used as predictors in a Gaussian Processes regression (GPR) with age as the outcome variable. (**b**) Model accuracy was assessed in a ten-fold cross-validation procedure, comparing brain-predicted age with original chronological age labels. (**c**) Model coefficients learned during training were then applied to the data from LBC1936 participants to make age predictions. (**d**) A metric to summarise the variation in predicted age was defined; the brain-predicted age difference (brain-PAD; predicted age—chronological age). LBC1936, Lothian Birth Cohort 1936; MRI, magnetic resonance imaging; WM, white matter.

The machine learning age-predictions methods using neuroimaging data are outlined in Figure 1. Briefly, T1-weighted MRI scans were segmented into grey matter (GM) and white matter (WM) before being normalised in common space using non-linear spatial registration. Once normalised, GM and WM images were concatenated and converted into a similarity matrix of training subjects’ data, which to predict chronological age in a Gaussian Process regression. Model accuracy was then validated using ten-fold cross-validation, comparing brain-predicted age with chronological age. The coefficients ‘learned’ from the full model (*N*=2001) were then applied to the test data (LBC1936, *N*=669) to make brain-based age predictions for these individuals. Brain-PAD (predicted age—chronological age) was then calculated and used for further statistical analysis.

### Ageing fitness measures

Five measures of ‘fitness’, or a healthy ageing phenotype,^30^ in older age were considered: walking speed (time to walk 6 metres), right-hand grip strength (measured by a dynamometer), lung function (forced expiratory volume in 1 s), cognitive function (fluid-type intelligence) and allostatic load.^31^ Allostatic load was derived from measures of: fibrinogen, triglyceride, high-density lipoprotein, low-density lipoprotein, total cholesterol, cholesterol high-density lipoprotein ratio, glycated haemoglobin, C-reactive protein, interleukin-6, body-mass index and blood pressure. All measures used in the present analysis were collected at the same time as the neuroimaging assessment.

### Mortality ascertainment

Mortality status was obtained via data linkage to the National Health Service Central Register, provided by the National Records of Scotland. The LBC1936 research team are routinely informed of participant deaths and cause of death approximately every 12 weeks. Most recent ascertainment was at approximately age 79 years (range 78.7–79.7 years), which was between 5.4 and 7.9 years after neuroimaging assessment.

### Molecular genetic biomarkers of ageing

Using whole blood samples, data for two candidate ageing biomarkers were generated. The ‘epigenetic clock’^3^ was used to calculate predictions of age based on DNA-methylation status at 450, 726 autosomal sites across the genome, as per the previously reported ‘Horvath’ protocol.^26, 32^ Leukocyte telomere length was measured using a protocol developed at University of Newcastle.^33^

## Results

### Chronological age can be predicted using neuroimaging

**Figure 2:**
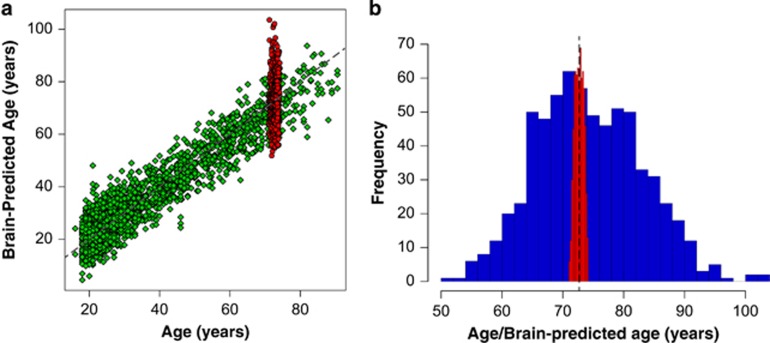
Brain-predicted age using structural neuroimaging in LBC1936. (**a**) Scatterplot showing the relationship between chronological age and brain-predicted age in the independent healthy cohort used as the training data (green diamonds) and the LBC1936 participants used as the test set (red circles). (**b**) Histogram showing the distributions of brain-predicted age (in blue) compared to the distribution in chronological age (in red). The substantially wider variability in brain-predicted age is evident. LBC1936, Lothian Birth Cohort 1936.

A machine-learning model (Gaussian Processes), trained on the brains of *N*=2001 healthy adults, aged 18–90 years, can accurately predict chronological age using T1-weighted MRI scans (Figure 2a). Cross-validation results gave a correlation between brain-predicted age and chronological age of *r*=0.94, (*P*<0.001, corrected after 1000 permutations) and explained 88% of the variance (*R*^2^). The mean absolute error of prediction was 5.02 years and the root mean square error was 6.31 years. This training stage validated our model of brain-predicted age, for use in predicting age with neuroimaging data collected in other samples.

### Older adults show marked variation in structural brain ageing

The model coefficients ‘learned’ from the training dataset were applied to T1-weighted MRI scans acquired from the LBC1936 participants (Table 1) to generate a brain-predicted age. At the time of scanning LBC1936 participants had a mean chronological age of 72.67 (s.d.=0.73) years and a mean brain-predicted age of 74.32 (s.d.=8.72) years. The mean absolute error of age prediction in the LBC1936 participants was 7.08 years and the root mean square error was 8.85 years. The variability in brain-predicted age was considerably greater than the variability in chronological age, reflecting marked individual differences in brain structure in participants aged approximately 73 (Figure 2b). As expected, brain-PAD scores did not correlate with chronological age (*r*=−0.01, *P*=0.79), indicating that deviations from healthy brain ageing (that is, having an older- or younger-appearing brain) were not related to underlying chronological age. Females’ brain-predicted ages were, on average, younger than their chronological age (mean (s.d.) brain-PAD=−1.29 (7.87) years), whereas males’ were older (mean (s.d.) brain-PAD=4.29 (8.58) years). This sex difference was statistically significant (Wilcoxon rank-sum test, W=35431, *P*<0.001), hence sex was included as a covariate in all further analyses.

### Early mortality is associated with older-appearing brains

**Figure 3:**
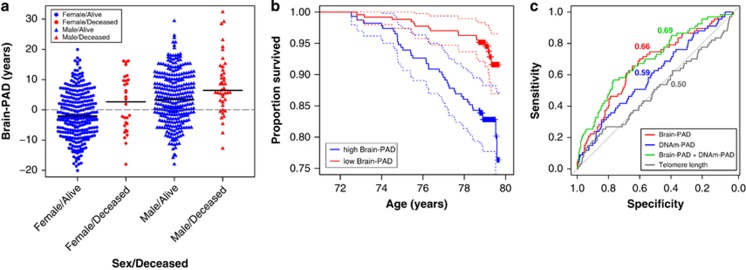
Association of mortality with brain-predicted age difference and DNA-methylation-predicted age difference. (**a**) Grouped scatterplot showing the relationship between brain-predicted age difference (brain-PAD) score (i.e., brain-predicted age—chronological age) and mortality (alive=blue, dead=red), sub-divided by sex (female=circle, male=triangle). Mortality status was determined ~6 years after MRI assessment. Horizontal black lines represent the median for each sub-group. (**b**) Kaplan–Meier plot of right-censored survival data since MRI assessment. The two lines represent a tertile split based on brain-PAD score, with highest 33.3% being classed as high brain-PAD (red line) indicating increased brain ageing and the lowest 33.3% (low brain-PAD, blue line) indicating reduced brain ageing. Crosses indicate censoring points (i.e. age at last survival ascertainment). Dotted lines represent the 95% confidence intervals. (**c**) Figure depicts the receiver operator characteristic (ROC) curves for four contrasting, nested, survival models. All models used mortality status as the response variables. The predictor variables were Brain-PAD (red line, model 1), DNAm-PAD (blue line, model 5), Brain-PAD+DNAm-PAD (green line, model 4), Telomere length+Brain-PAD+DNAm-PAD (grey line, model 3). The areas under the curve (AUC) are coloured-coded and appear next to each ROC curve. MRI, magnetic resonance imaging.

Having a higher brain-PAD score (that is, a brain that appears older than one’s chronological age) was significantly associated with mortality before the age of 80 (*P*<0.001); up to seven years after neuroimaging assessment. Mean brain-PAD score for deceased males (*N*=43) and females (*N*=30) was 8.13 (s.d.=9.52) and 2.07 (s.d.=9.27) years, respectively, compared to 3.76 (s.d.=8.32) and −1.64 (s.d.=7.65) years for surviving males and females (Figure 3). The relationship between mortality risk and brain-PAD was tested using Cox proportional hazards regression analysis, adjusting for age and sex. Survival was ascertained up to 7.9 years post-neuroimaging, and survival duration was right-censored for surviving individuals based on days between neuroimaging assessment and mortality ascertainment. Each extra year of brain-predicted age (that is, having a brain-PAD score of +1) resulted in a 6.1% relative increase in the risk of death between age 72 and 80 (hazard ratio (HR)=1.061, 95% confidence interval (CI)=1.031, 1.091, *P*<0.001). The assumptions of proportional hazards were met by the model. An illustrative Kaplan–Meier plot using the upper and lower tertiles of brain-PAD scores in LBC1936 participants is shown in Figure 2b. The influence of other variables previously related to mortality in this sample was considered in a fully-adjusted model, as per Marioni and colleagues.^26^ These were: Moray House Test IQ-type score at age 11, paternal social class (five-point scale), years of full-time education, *APOE* e4 carrier status, smoking status (never, ex-smoker, current smoker), and self-reported hypertension, diabetes and cardiovascular disease. Brain-PAD remained significantly associated with mortality risk in this fully-adjusted model, with a slight attenuation of the effect size (HR=1.051, 95% CI=1.020, 1.083, *P*<0.001; Supplementary Table S1).

### Variability in apparent brain-ageing relates to physical and mental fitness

Brain-PAD score was also significantly related to a number of measures that reflect characteristics of physical and mental fitness in older age using linear regression (Supplementary Table 2). An older-appearing brain, as indicated by a higher brain-PAD score, was significantly associated with lower fluid cognitive performance (standardised beta=−0.121, *P*=0.007), weaker grip strength (standardised beta=−0.060, *P*=0.020), poorer lung function (standardised beta=−0.072, *P*=0.020) and slower walking speed (standardised beta=0.133. *P*=0.004). Higher brain-PAD score was also associated with higher allostatic load (standardised beta=0.097, *P*=0.020), a composite measure of biological and physiological parameters, designed to reflect biological ‘wear-and-tear’ accumulated over a lifetime of stress adaptation. Reported *P*-values were corrected for five tests using a 5% false discovery rate.

### Brain-PAD is not related to the prevalence of morbidity

Next, we examined the relationship between brain-PAD and the presence of self-reported cardiovascular disease, stroke, and diabetes. LBC1936 participants reported the following prevalence of disease: cardiovascular disease=26.9% (*N*=180), diabetes=10.2% (*N*=68) and a history of stroke=6.9% (*N*=46). After adjusting for sex, there was no significant association between brain-PAD score and cardiovascular disease (*P*=0.08), diabetes (*P*=0.14) or stroke (*P*=0.85).

### Brain-PAD is not related to childhood IQ, life-course social factors or *APOE* e4 status

Brain-PAD was also not associated with potential life-course influences on ageing. Potential influences tested were: performance on the Moray House Test at age 11 (*P*=0.63), paternal social class (*P*=0.82), years of education (*P*=0.45), neighbourhood deprivation as indexed by the Scottish Index of Multiple Deprivation (*P*=0.45), and the presence of an *APOE* e4 allele (*P*=0.88).

### Brain-PAD and conventional neuroimaging measures in relation to survival

Brain-PAD was significantly correlated (positively or negatively) with: GM, normal-appearing white matter, cerebrospinal fluid (CSF) and WM hyperintensity volume, whole brain cortical thickness, fractional anisotropy and mean diffusivity (Supplementary Figure 1). When combining brain-PAD with these imaging measures to predict outcomes, brain-PAD contributed unique variance (determined using hierarchical variance partitioning) to each linear regression model (Supplementary Table 2). Although brain-PAD was not always the greatest contributor of unique variance to outcome prediction, this analysis indicates that brain-PAD can add complementary information to models of age-related outcomes, over and above that gained from conventional neuroimaging measures. Further, we assessed whether GM, normal-appearing white matter and CSF volume were associated with survival. Cox regression analyses, adjusted for age and sex, indicated that GM and CSF volume (in ml) were associated with survival (GM: HR=0.991, 95% CI=0.984, 0.998, *P*=0.007; CSF: HR=1.012, 95% CI=1.007, 1.017, *P*<0.001), where a having 1 ml lower GM or 1 ml higher CSF volume was associated with a 1% increase in mortality risk. Normal-appearing white matter volume was not associated with mortality risk (*P*=0.54). We then compared the predictive value of linear combinations of brain-PAD with GM and CSF volume in Cox regression models (that is., brain-PAD+GM volume, brain-PAD+CSF volume, brain-PAD+GM volume+CSF volume). Brain-PAD significantly related to survival in the paired combined models (*P*<0.05), indicating that it independently explained some variance relating to survival, when combined with GM volume or with CSF volume separately. However, when included alongside both GM volume and CSF volume, brain-PAD was no longer a significant predictor of survival (*P*=0.12), while the volumetric measures remained significant (GM: *z*=−3.78, *P*<0.001; CSF: *z*=4.56, *P*<0.001). For full details see Supplementary Table 3.

### Brain-PAD combined with DNA-methylation ‘age’ improves survival modelling

Molecular genetic ageing biomarkers have also been proposed, hence we compared brain-PAD with DNA-methylation status and leukocyte telomere length. DNA-methylation (DNAm) age was predicted using Horvath’s ‘epigenetic clock’ method,^3^ in *N*=620 (female=290, male=330) participants, who had undergone neuroimaging assessment. Mean DNAm-predicted age was 69.3 (s.d.=6.2) years. Mean DNAm-predicted age difference (DNAm-PAD) was −3.4 (s.d.=6.1) years. Mean DNAm-PAD was similar for males (−3.2, s.d.=5.8) and females (−3.7, s.d.=6.4), with no statistically significant sex difference (W=44803, *P*=0.17). There was no association between the DNAm-predicted age and brain-predicted age (rho=0.001, *P*=0.98) or between brain-PAD and DNAm-PAD (rho=−0.007, *P*=0.85). Regarding telomere length, data were available in *N*=653 participants (female=309, male=344) with neuroimaging data. Telomere mean (s.d.) length was 3982.5 (711.7) base-pairs. Telomere length in females was 4045.5 (635.2) base-pairs, while for males it was 3912.3 (783.3) base-pairs, which was significantly different (W=45386, *P*=0.001). There was no significant association between telomere length and brain-PAD (rho=0.04, *P*=0.31) or brain-predicted age (rho=0.05, *P*=0.23).

Combining DNAm-PAD and telomere length with brain-PAD in a multivariate Cox regression, adjusted for age and sex, significantly predicted survival (*N*=608, deceased *N*=67, *P*<0.001). Within this model, brain-PAD (HR=1.07, 95% CI=1.04, 1.11, *P*<0.001) and DNAm-PAD (HR=1.06, 95% CI=1.02, 1.10, *P*<0.001) were significant contributors to the prediction, while telomere length was not (*P*=0.97). A separate model using DNAm-PAD alone also significantly predicted survival (HR=1.06, 95% CI=1.02, 1.09, *P*<0.001); however, this explained significantly less variance than a model using brain-PAD alone (AUC=0.59 vs brain-PAD alone AUC=0.66, *P*<0.001). The combined model using brain-PAD and DNAm-PAD explained significantly more variance than either variable alone (combined model AUC=0.69 vs brain-PAD alone AUC=0.66 vs DNAm-PAD alone AUC=0.59, *P*<0.001; see Figure 2c, Supplementary Table 4). This was also the case for the fully-adjusted model covarying for potential influences on mortality risk. Prediction of ageing fitness measures was not improved when combining brain-PAD and DNAm-PAD or brain-PAD and telomere length.

## Discussion

Here we showed that a neuroimaging-based marker of brain ageing is associated with a greater risk of death and poorer physical and cognitive fitness in a large cohort of older adults. Furthermore, we demonstrate that combining biological age predictions generated from neuroimaging and DNA-methylation status data increases the accuracy of survival modelling. At ~73 years of age, we found that people with brains that appeared older than their chronological age had, in addition to greater mortality risk: weaker grip strength, poorer lung function, slower walking speed, lower fluid general intelligence, and had been exposed to greater allostatic load (a biological measure intended to summarise the cumulative effects of lifetime biological ‘wear and tear’). The relationship between brain-PAD and survival was independent of life-course influences on mortality including: education, social class, childhood IQ, carrying an *APOE* e4 allele or the presence of age-associated illness. Furthermore, these factors were themselves not significantly associated with brain-PAD in this sample.

To the best of our knowledge, this is the first demonstration that a neuroimaging-derived age prediction is associated with higher mortality risk. Such measures have been used in clinical samples, showing increased apparent brain age following traumatic brain injury^20^ and in individuals with mild cognitive, a key risk factor for Alzheimer’s Disease.^18^ Higher levels of exercise^21^ and meditation^22^ have been associated with lower brain age in the healthy population, but the link with mortality is novel. This is crucial, as it supports the use of MRI as a screening tool to help identify people at greater risk of general functional decline and mortality during ageing. Brain-PAD has the potential to be estimated in large numbers of people, as MRI is collected routinely in clinical settings. The success of projects like UK Biobank^34^ shows that acquiring MRI on a very large scale is feasible given the appropriate infrastructure.

The combination of DNA-methylation-predicted age and neuroimaging-predicted age is also novel. We found that, while brain-predicted age significantly out-performed DNA-methylation predicted age, there is greater added value gained when combining these two approaches to predict survival. Previously, ‘DNA-methylation age’ has been related to mortality and ageing fitness,^26, 32^ and in various clinical contexts including HIV,^35^ Down’s Syndrome^36^ and obesity.^37^ Interestingly, brain-PAD and DNAm-PAD were not correlated, yet both related to survival independently and improved survival prediction when analysed in combination, thus provided complementary information. This demonstrates that contrasting approaches to estimating age biologically can be integrated to predict clinically-relevant outcomes. Seemingly, epigenetic ageing in leukocytes and ageing of brain structure are occurring independently, perhaps evidence for a ‘mosaic’ of ageing,^24^ where biological ageing occurs at different rates in different systems or compartments within an individual. This motivates further research that combines independent measures of biological ageing to develop a more global ageing biomarker, which may further improve predictions of survival.

Other neuroimaging measures have previously been associated with mortality in older adult population cohorts. These include WM hyperintensities in adults aged 70–82 years,^38^ regional volume reductions at age 85 years^39^ and whole brain volume at 78–85,^40^ 66–90^41^ and 60–90 years.^42^ Visual assessment of infarcts, WM hyperintensities and atrophy also predicted mortality 6 months after a stroke.^43^ This research supports the idea that the brain plays a central role in the ageing process and is sensitive to the cumulative damage that accrues throughout life and increases mortality risk. That we can predict mortality before the age of 80 using neuroimaging assessment at approximately age 73, fits with these previous reports.

Interestingly, when combining brain-PAD with GM and CSF volume in a Cox regression, brain-PAD no longer significantly predicted survival. This indicates that the survival-related variance in brain-PAD can be captured using more conventional volumetric measures. While brain-PAD did incrementally improve survival prediction over individual volumetric measures, our results indicate that using a combination of GM and CSF volume is potentially a strong biomarker of mortality. Nevertheless, these volumetric measures appear less suitable as an ageing biomarker *per se*, as a linear model of GM, WM and CSF volume explained only 66% of variance in chronological age (mean absolute error=8.30 years, root mean square error=10.53) in the training dataset, compared with 88% using brain-predicted age. This demonstrates that in the context of developing an ageing biomarker, there is benefit in using a machine-learning approach to analyse high-dimensional voxelwise T1-MRI data, compared to macroscopic volume measurements. Future steps to further improve models of brain ageing and derived ageing biomarkers could incorporate additional imaging modalities at the modelling stage. This should capture further age-associated changes including WM hyperintensities using FLAIR-MRI, altered WM microstructure using diffusion-MRI and beta-amyloid deposition using positron emission tomography.

A key medical research goal is to identify reliable predictors of mortality, proxy measures of underlying pathological processes that increase mortality risk. For example, grip strength has been robustly associated with mortality,^44, 45^ and is thought to be a proxy of the musculo-skeletal system. Similarly, brain-PAD may be a general reflection of CNS health. Grip strength measures do not necessarily require a direct causal link with cardiovascular or all-cause mortality to be clinically useful;^46^ the same could apply to brain-PAD. Moreover, the relevance of brain-predicted age for health is intuitively straightforward. Already, the UK National Health Service encourages people to complete a cardiovascular health question to determine their ‘heart age’ (www.nhs.uk/Conditions/nhs-health-check/Pages/check-your-heart-age-tool.aspx). By analogy, ‘brain age’, or a global ‘biological age’, could be used in public health settings to convey complex information to patients in readily comprehendible terms.

Brain-PAD related to all measures of ageing fitness. This suggests that our measure of brain ageing relates to some more general facets of physiological ageing. Along with grip strength, all these measures (lung function, gait speed, cognitive function and allostatic load) have been previously associated with mortality.^47, 48, 49, 50^ As proxies for systemic health (for example, musculo-skeletal, respiratory, nervous, circulatory), they appear to relate to a common aspect of more general health of the whole body, likely due to the interactions between different human biological systems. However, there also seems to be unique variance in the relationship of these measures with mortality. This is supported by our finding that survival modelling accuracy was improved when including multiple ageing fitness measures alongside brain-PAD. Notably, brain-PAD remained the strongest predictor in this combined model, which justifies further research into the clinical applications of neuroimaging-based predictors of mortality.

Our study has some strengths and weaknesses, particularly relating to the cohorts under study. The sample size for both training and test sets is relatively large. One potential limitation is the multiple sources of training data. Comprehensive demographic data were not available on all these individuals. However, individuals in this sample were screened according to various criteria to ensure that were free of manifest neurological, psychiatric or major medical health issues. The LBC1936 is well-characterised, allowing a broad exploration of relationships with brain-predicted ageing, particularly the follow-up to assess mortality. The limited age range of LBC1936 participants is a strength in that it eliminates the important confounding effect of chronological age, but it may limit generalisations to other age groups. However, this point in the life course is a timely juncture to assess brain ageing as individual differences have had time to accumulate though are unlikely to be widely confounded by manifest neurodegenerative disease. The current analysis was cross-sectional; therefore, we cannot determine whether the relationship between brain-predicted age and mortality risk varies with age or where on the trajectory of atrophy an individual is. The on-going nature of the LBC1936 study will allow future analysis of longitudinal data to determine whether trajectories of brain ageing are better indicators of future health outcomes than cross-sectional measures. In addition, we only assessed all-cause mortality, which limits speculation about causal relationships between brain structural alterations and specific mortality causes, such as cardiovascular or neurological causes of death. Finally, the LBC1936 participants were not fully representative of the population from which they were drawn. Compared to the full population who sat the cognitive test at age 11, LBC1936 participants had higher cognitive ability,^27^ and in later life were likely to be healthier than their peers in the general population. This may have been due to selection effects seen in most studies of ageing. That our sample might have missed individuals with particularly poor health or high frailty. Hence we might have underestimated some of the effects reported here, as a small number of individuals with worse performance on measures of ageing fitness may not have been included.

The difference between neuroimaging-predicted age and chronological age is associated with survival in a large sample of older adults and relates to measures of cognitive and physical fitness. Moreover, combining age-predictions from DNAm and neuroimaging data increased the accuracy of survival modelling. This study provides evidence that neuroimaging data can be used to construct a viable ageing biomarker, and potentially provides important prognostic information, particularly in combination with complementary epigenetic ageing data. A global biomarker of ageing has the potential to screen for asymptomatic individuals who are experiencing adverse ageing and thus are at increased risk of future ill-health and could be used as a surrogate outcome measure in clinical trials of neuroprotective treatments and anti-ageing therapeutics.

## Figures and Tables

**Table 1:** Lothian Birth Cohort 1936 characteristics

	*All*	*Male*	*Female*
*N*	669	352	317
Age	72.67 (0.73)	72.63 (0.71)	72.72 (0.74)
Mini-mental state examination (median (IQR))	29 (2)	29 (2)	29 (2)
Brain-predicted age	74.32 (8.72)	76.92 (8.64)	71.43 (7.88)
Brain-PAD	1.65 (8.71)	4.29 (8.58)	−1.29 (7.87)
g_f_	0.03 (0.98)	0.01 (1.05)	0.06 (0.90)
Grip strength	28.79 (9.33)	35.38 (6.71)	21.45 (5.63)
FEV_1_ (l)	2.34 (0.68)	2.72 (0.62)	1.92 (0.44)
6 metre walk time (s)	4.29 (1.21)	4.09 (1.11)	4.51 (1.27)
Allostatic load	−0.03 (0.99)	0.09 (0.95)	−0.15 (1.02)
Deceased (*N*)^a^	73	43	30

Abbreviations: brain-PAD, brain-predicted age difference; FEV, forced expiratory volume in one second; *g*_*f*_, fluid type general intelligence; IQR, inter-quartile range.

a Mortality was ascertained between 5.4 and 7.9 years after neuroimaging assessment.

Values reported are mean (s.d.) unless otherwise specified.
