# Supplementary material for: Brain age predicts mortality
Source: Mol Psychiatry. 2017 Apr 25;23(5):1385–92. doi: 10.1038/mp.2017.62 (PMC5984097; doi:10.1038/mp.2017.62)
Supplement: Supplementary Information [file mp201762x1.docx]

Supplementary Information

## Supplementary Methods

## Participants - Lothian Birth Cohort of 1936

The Lothian Birth Cohort of 1936 (LBC1936) is a longitudinal study of ageing, especially cognitive ageing^27, 28^. Most of the study members had participated in the Scottish Mental Survey of 1947 (SMS1947), when almost all children born in 1936 and attending school in Scotland completed a test of general cognitive ability, the Moray House Test, on June 4^th^ 1947. Individuals born in 1936 and were living in the Lothian region of Scotland in older age were subsequently contacted and invited to participate in LBC1936. In total, 1,091 people were recruited at Wave 1 (mean age about 70 years) with further follow-up waves at mean ages of about 73, 76, and 79 (in progress at the time of writing) years. Extensive phenotypic data have been collected, including blood biomarkers, neuroimaging, cognitive assessments, and psycho-social, lifestyle, genetic, epigenetic, physiological and health measures.

At Wave 2, 866 (male = 448 and female = 418) participants returned at a mean age of 72.5 (SD = 0.7) years. Of these, 700 underwent brain magnetic resonance imaging (MRI), resulting in 669 (352 = male, 317 = female) usable 3D T1-weighted MRI scans for the present analysis. The brain imaging protocol has been described in detail^29^. Ethical approval for the LBC1936 was obtained from the Multi-Centre Research Ethics Committee for Scotland (MREC/01/0/56) and the Lothian Research Ethics Committee (LREC/2003/2/29). Written informed consent was obtained from all subjects.

## Participants - Training cohort for brain-predicted age model

A further 2,001 healthy individuals (age mean = 36.95 ± 18.12 years; age range = 18-90 years; males = 1,016; females = 985) comprised the brain age prediction training cohort. These data were drawn from publicly-available data repositories (Supplementary Table 4) and represent either the healthy controls from case-control studies of disease or healthy individuals from large-scale brain mapping and atlas generation studies. These studies include a variety of different scanner vendors (Siemens, GE, Philips), different field strengths (1.5T, 3T) and T1 acquisition protocols (MPRAGE, T1-FFE, SPGR), with differing voxels sizes. All participants were screened according to local study protocols to ensure that they were free of neurological and psychiatric disorders, had no history of head trauma and other major medical conditions. Ethical approval for each initial study and subsequent data-sharing was verified per repository.

## Ageing fitness measures

Five measures of ‘fitness’, or a healthy ageing phenotype^30^, in older age were considered: walking speed, grip strength, lung function, cognitive function and allostatic load^31^. All measures used in the present analysis were collected at Wave 2.

Walking speed was measured as the time to walk 6 meters in seconds. Grip strength was assessed in the right hand three times using a North Coast Hydraulic Hand Dynamometer (JAMAR) with the best measure being recorded. Lung function was measured as the forced expiratory volume, in liters, in one second (FEV_1_) based on the highest score from three tests with a Micro Medical Spirometer.

Cognitive function was indexed by a measure of general fluid-type intelligence (*g_f_*), derived using a previously published procedure^32^. G*_f_* was derived from six non-verbal tests of cognitive function from the Wechsler Adult Intelligence Scale-III: letter-number sequencing and digit span backwards (working memory), matrix reasoning (non-verbal reasoning), block design (constructional ability), and digit symbol coding and symbol search (processing speed). As previously outlined^33^, the first unrotated component was extracted from a principal components analysis to represent this underlying latent construct (explaining 52% of the variance).

Allostatic load is a composite measure reflecting the cumulative physiological ‘wear-and-tear’ placed on a biological system during ageing. Using our previously defined confirmatory factor analysis (CFA) approach^31^, allostatic load was computed at LBC1936 Wave 2, based on 10 biomarkers that represent different contributing factors. These were: fibrinogen, triglyceride, high-density lipoprotein (HDL) and low-density lipoprotein (LDL), total cholesterol, cholesterol-HDL ratio, glycated hemoglobin, C-reactive protein, interleukin-6, body-mass index and mean systolic blood pressure (SBP) and diastolic blood pressure (DBP). Allostatic load scores were calculated for each participant by taking the mean of the first-order factor loadings across the different measures, based on CFA in which the variance of each latent variable was fixed at 1.

## Mortality ascertainment

Mortality status was obtained via data linkage to the National Health Service Central Register, provided by the National Records of Scotland. The LBC1936 research team are routinely informed of participant deaths and cause of death on approximately a 12-weekly basis. Most recent mortality ascertainment was at approximately age 79 years (range 78.7-79.7 years), which was between 5.4 and 7.9 years after Wave 2 neuroimaging assessment.

## *APOE* Genotyping

Analysis of the apolipoprotein E gene (*APOE*) used DNA isolated from whole blood. The target sequences for single nucleotide polymorphisms (SNPs) rs7412 and rs429358 were genotyped using TaqMan technology ([Invitrogen website, 2012](http://www.invitrogen.com/site/us/en/home/brands/taqman.html)) for polymerase chain reaction. These two SNPs form the *APOE* ε2/ε3/ε4 haplotype, commonly referred to as *APOE* genotype, with ε4 representing the dementia risk genotype. The distribution of genotypes was confirmed as not deviating from Hardy-Weinberg equilibrium (*P* = 0.66).

## Childhood IQ, life-course social factors

Childhood IQ was measured using data from the Moray House Test, conducted in 1947 as part of the Scottish Mental Survey^27^. Neighborhood deprivation was assessed at Wave 1 with the Scottish Index of Multiple Deprivation ([www.scotland.gov.uk/topics/statistics/simd](http://www.scotland.gov.uk/topics/statistics/simd)). Paternal social class was also assessed at Wave 1 and was rated on a 5-point scale, based on estimates of paternal income and years of education.

## DNA-methylation age prediction

The protocol for calculating DNA methylation age followed the ‘epigenetic clock’ method, devised by Horvath^3^. These data were obtained for the LBC1936 as has been previously reported^26, 32^. Briefly, DNA was extracted from whole blood samples, following standard procedures at the Wellcome Trust Clinical Research Facility, Western General Hospital, Edinburgh (https://www.wtcrf.ed.ac.uk/Home). DNA samples were hybridized to the 12 sample Illumina Human-Methylation450BeadChips using the Infinium HD Methylation protocol (Illumina, San Diego, CA, USA). Quality control was performed and background corrected probes at 450,726 CpG sites were used to calculate DNA methylation age, according to Horvath’s epigenetic clock^3, 26, 32^.

## Telomere length

Telomere length was determined as previously reported^34^ at the University of Newcastle. This involved the extraction of DNA from leukocytes in whole blood, followed by quantitative polymerase chain reaction (qPCR), using an Applied Biosystems (Pleasonton, CA, USA) 7900HT Fast Real Time PCR machine. This was done to determine the abundance of telomeric template compared to the GAPDH gene. Internal control samples were run within each plate. These internal controls have a known absolute telomere length. The relative ratio values (telomere starting quantity/GAPDH starting quantity) of these cell lines were then used to generate a regression model so that values of relative telomere length for the experimental samples could be converted into absolute telomere lengths. Measurements were performed four times for robustness.

## Neuroimaging: data acquisition

Structural MRI data were obtained at Wave 2 with a GE Signa Horizon HDxt 1.5T scanner (General Electric, Milwaukee, WI, USA) at the Brain Research Imaging Centre, Edinburgh, using a self-shielding gradient set with maximum gradient strength of 33 m/Tm and an 8-channel phased-array head coil^29^. High-resolution 3D fast spoiled gradient echo (FSPGR) T1-weighted (TR = 9.8, TE = 4, inversion time = 500 ms) volume scans were acquired in the coronal plane with 160 slices at 1.3 mm thickness and an in-plane resolution of 1 mm x 1 mm. Three-dimensional structural images for the training set were acquired using various parameters and field strengths (1.5T and 3T), according to local study protocols (see Supplementary Table 4).

## Neuroimaging: pre-processing

All structural images were pre-processed using SPM12 (University College London, London, UK). Images were bias corrected and segmented into GM, WM and cerebrospinal fluid (CSF) using SPM *Segment*. Visual quality control was carried out at this stage to ensure accuracy of image segmentation. Segmented images for GM and WM were then non-linearly registered to a custom template, based on the training dataset, using SPM *DARTEL*^35^. Finally, images were affine registered to MNI152 space (1.5mm^3^) and resampled using modulation to retain the volumetric information and smoothed with a 4mm Gaussian kernel. A summary measures of intracranial volume (ICV) was also generated.

## Neuroimaging: Machine learning for brain-predicted age

Brain-predicted ages were generated following a previously described protocol^20^, using the Pattern Recognition for Neuroimaging Toolbox (PRoNTo v2.0, [www.mlnl.cs.ucl.ac.uk/pronto](http://www.mlnl.cs.ucl.ac.uk/pronto/)). First, a model of healthy brain ageing was defined using brain volumetric maps from a training dataset (N = 2001). Spatially normalized images were converted to vectors and the resulting GM and WM vectors were concatenated for each individual. A linear kernel representation of these data was derived by calculating an *N* x *N* similarity matrix, where each point in the matrix was the dot product of two subjects’ image vectors. This step retains all the original image variance in a much sparser representation, greatly reducing subsequent computation time. A Gaussian Processes regression model was defined, with chronological age as the dependent variable and the image data (in similarity matrix form) as the independent variables. This model thus represents the correspondence between age and multivariate voxelwise measures of brain volume. Predictions on all subjects were generated using ten-fold cross-validation, whereby the data were randomly divided into 10 folds, each comprising 10% of the subjects. The model was then re-trained using 9 of the data folds and age predictions were made on the ‘left-out’ fold. This procedure was iterated so that all folds were left-out in turn, resulting in unbiased (i.e. independent) age predictions for each subject. Model accuracy was expressed as the correlation between age and predicted age (Pearson’s *r*), total variance explained (R^2^), mean absolute error (MAE) and root mean squared error (RMSE). Statistical significance of this model was assessed using permutation testing (n = 1,000). Cross-validation in the training set indicated that the model was able to accurately predict age (*r* = 0.938, R^2^ = 0.88, MAE = 5.01, RMSE = 6.31), based on combined GM and WM volume images (permutation corrected *P* = 0.001). However, a proportional bias was detected whereby chronological age correlated with prediction error (*r* = -0.25, *P* < 0.001). To remove this bias from future predictions, an additional adjustment was made based on the slope and intercept of a linear regression model of chronological age against brain age.

Next, the coefficients from the full model of healthy brain age (trained on N = 2001) were applied to the test data (i.e. LBC1936 participants, N = 669), generating unbiased estimates of age based on GM and WM volume images. Finally, brain-predicted age difference (brain-PAD) scores were calculated for each test subject by subtracting chronological age from predicted age. Hence, a positive brain-PAD score indicates that the individual’s brain is predicted to be ‘older’ than their chronological age. Brain-PAD scores were subsequently used for further analysis to index relative brain ageing.

**Neuroimaging: code availability**

The MATLAB code used to generate the neuroimaging results is available from the open access software packages SPM and PRONTO, details are above.

## Neuroimaging: conventional structural measures

To compare brain-PAD with more commonly used neuroimaging measures of brain structure, the following MRI data were acquired: T1-weighted, T2-weighted, diffusion-weighted, FLAIR and T2* axial imaging. These were then processed as previously reported^13, 29, 36, 37^ and generated the following metrics: GM volume, normal-appearing WM volume, whole brain volume, CSF volume, WM hyperintensity volume (all volumes as a ratio of ICV), mean cortical thickness, and whole brain WM fractional anisotropy and whole brain WM mean diffusivity factors, calculated as previously outlined^38^. These data were available in 97.9% of the cohort (N = 655).

## Statistical analysis

Using brain-PAD scores derived from structural neuroimaging of the LBC1936 participants, further statistical analysis was carried out using R v3.2.3. To explore the association between brain-PAD and measures of fitness in older age, linear regression models were defined for each fitness variable (g_f_, grip strength, FEV_1_, 6-m walk time, allostatic load). Each model had the fitness variable as the outcome variable, brain-PAD score as the predictor variable with age and sex as covariates. Further covariates were added for specific models: i.e., height (grip strength, FEV_1_, 6-m walk time) and current smoking status (FEV_1_). The relationships between predicted brain-age and telomere length and DNA methylation age were examined using a Spearman’s rank-order correlation. Group comparisons based on sex were conducted using a Wilcoxon rank sum test. To determine unique variance contributed by different neuroimaging measures to outcome prediction, a hierarchical partitioning of variance was carried out using R. All eight neuroimaging measures were included as predictors, along with age and sex, and separate regression models were run for fluid cognition, grip strength, lung function, walking speed and allostatic load.

To investigate the relationship between mortality and brain-PAD, a survival analysis using a Cox proportional hazards regression was conducted using the R package ‘survival’^39^. Analysis used the most recent mortality data available, at approximately age 79 years. Data were right censored based on age (in days) at most recent assessment, with brain-PAD (in years) as the unit of exposure. Age (in days) at time of scanning (i.e. Wave 2) and sex were used as covariates in a basic model. Further, to explore other potential influences on mortality, and whether they might be confounders and/or mediators of any Brain-PAD-mortality association, additional variables were included in a full model. These were Moray House Test IQ-type score at age 11 years^40^, paternal social class (5-point scale), years of full-time education, *APOE* e4 carrier status, smoking status (never, ex-smoker, current smoker), and self-reported hypertension, diabetes and cardiovascular disease. To illustrate the relationship between PAD score and age at death, data were split into tertiles based on PAD score and the upper and lower tertiles used in a Kaplan-Meier curve, adjusting for age and sex.

## Supplementary Table 1. Survival modelling details

| Model | Hazard Ratio | 95% Confidence Interval | z | *P* | Model R^2^ |
| --- | --- | --- | --- | --- | --- |
| 1) Brain-PAD only (N = 669, 73 deceased) |  |  |  |  | 0.032 |
| Brain-PAD | 1.061 | [1.031, 1.091] | 4.13 | <0.001 |  |
| 2) Brain-PAD fully-adjusted for potential influences on mortality (N = 636, 69 deceased) | | | | | 0.077 |
| Brain-PAD | 1.051 | [1.020, 1.083] | 3.27 | 0.001 |  |
| Education (years) | 1.054 | [0.832, 1.336] | 0.44 | 0.66 |  |
| Smoking status (ex-smoker) | 0.386 | [0.207, 0.721] | -2.99 | 0.002 |  |
| Smoking status (never smoked) | 0.179 | [0.087, 0.366] | -4.70 | <0.001 |  |
| Moray House Test 1947 | 0.996 | [0.980, 1.012] | -0.51 | 0.61 |  |
| APOE e4 allele | 1.204 | [0.681, 2.130] | 0.64 | 0.52 |  |
| Diabetes | 2.050 | [1.122, 3.747] | 2.33 | 0.02 |  |
| Cardiovascular disease | 1.159 | [0.691, 1.943] | 0.56 | 0.58 |  |
| High blood pressure | 1.253 | [0.767, 2.048] | 0.90 | 0.37 |  |
| 3) Brain-PAD, DNAm-PAD, Telomere length  (N = 608, 67 deceased) | |  |  |  | 0.058 |
| Brain-PAD | 1.074 | [1.043, 1.107] | 4.71 | <0.001 |  |
| DNAm-PAD | 1.059 | [1.024, 1.096] | 3.33 | <0.001 |  |
| Telomere length | 1.000 | [0.999, 1.000] | -0.04 | 0.97 |  |
| 4) Brain-PAD, DNAm-PAD, (N = 620, 68 deceased) | |  |  |  | 0.054 |
| Brain-PAD | 1.069 | [1.039, 1.099] | 4.58 | <0.001 |  |
| DNAm-PAD | 1.056 | [1.022, 1.093] | 3.19 | <0.001 |  |
| 5) DNAm-PAD only |  |  |  |  | 0.022 |
| DNAm-PAD | 1.056 | [1.022, 1.092] | 3.22 | <0.001 |  |
| 6) Brain-PAD, DNAm-PAD fully adjusted for potential influences on mortality  (N = 591, 64 deceased) | | | | | 0.090 |
| Brain-PAD | 1.058 | [1.026, 1.092] | 3.58 | <0.001 |  |
| DNAm-PAD | 1.039 | [0.997, 1.082] | 1.83 | 0.07 |  |
| Education (years) | 0.983 | [0.764, 1.265] | -0.13 | 0.90 |  |
| Smoking status (ex-smoker) | 0.405 | [0.208, 0.786] | -2.67 | 0.007 |  |
| Smoking status (never smoked) | 0.201 | [0.095, 0.426] | -4.20 | <0.001 |  |
| Moray House Test 1947 | 0.998 | [0.983, 1.014] | -0.23 | 0.82 |  |
| APOE e4 allele | 1.121 | [0.629, 1.999] | 0.39 | 0.70 |  |
| Diabetes | 2.015 | [1.077, 3.770] | 2.19 | 0.03 |  |
| Cardiovascular disease | 1.352 | [0.785, 2.330] | 1.09 | 0.28 |  |
| High blood pressure | 1.286 | [0.765, 2.163] | 0.95 | 0.34 |  |

Results are from Cox proportional hazard regression models of survival duration. All the above models included age at scan and sex as additional predictor variables.

## Supplementary Figure 1. Brain-PAD correlates with conventional structural neuroimaging measures

Supplementary Figure 1. shows Pearson’s correlations between brain-PAD and seven conventional structural neuroimaging measures, in the LBC1936 participants (N = 482 had complete neuroimaging data). Brain-PAD = brain-predicted age difference, GM = grey matter, NAWM = normal appearing white matter, CSF = cerebrospinal fluid, WMH = white matter hyperintensities, ICV = intra-cranial volume.

## Supplementary Table 2. Hierarchical partitioning of neuroimaging variables when predicting ageing fitness measures

| Neuroimaging variables | *g_f_* | Grip strength | FEV_1_ (l) | 6 metre walk time (s) | Allostatic load |
| --- | --- | --- | --- | --- | --- |
| Brain-PAD | 3.35% | 36.34% | 19.10% | 8.71% | 5.21% |
| GM:ICV ratio | 9.13% | 7.91% | 4.61% | 21.30% | 11.70% |
| NAWM:ICV ratio | 37.20% | 4.28% | 7.30% | 9.51% | 3.83% |
| CSF:ICV ratio | 16.27% | 15.18% | 9.23% | 9.79% | 24.26% |
| WMH:ICV ratio | 14.88% | 11.15% | 10.29% | 14.72% | 3.46% |
| Whole brain cortical thickness | 23.68% | 20.87% | 36.99% | 19.79% | 33.15% |
| Fractional Anisotropy | 10.00% | 1.71% | 6.79% | 21.47% | 10.83% |
| Mean Diffusivity | -14.51%* | 2.55% | 5.69% | -5.30%* | 7.56% |
| Total variance explained (adjusted R^2^) | **0.08** | **0.59** | **0.44** | **0.12** | **0.09** |

Results of hierarchical partitioning of variance from multiple linear regression models using eight neuroimaging variables to predict ageing fitness measures (N = 482 had complete neuroimaging data). Values represent percentage of variance explained in the model that is uniquely attributable to each neuroimaging variable. The final row shows total variance explained by each joint model as a proportion of the variance in the outcome measure (i.e., adjusted R^2^). Brain-PAD = brain-predicted age difference, GM = grey matter, NAWM = normal appearing white matter, CSF = cerebrospinal fluid, WMH = white matter hyperintensities, ICV = intra-cranial volume. *g*_f_ = fluid cognitive performance. FEV = forced expiratory volume of air from the lungs in one second. Grip strength was the right hand. *Negative values indicate that this variable explains more variance in the joint model of all eight variables than it does alone, potentially acting to suppress noise in the joint model.

## Supplementary Table 3. Brain-PAD and brain volumes as predictors in survival models

| Model | Hazard Ratio | 95% Confidence Interval | z | *P* | Model R^2^ |
| --- | --- | --- | --- | --- | --- |
| **Brain-PAD only** |  |  |  |  | 0.033 |
| Brain-PAD | 1.061 | [1.031, 1.091] | 4.11 | <0.001 |  |
| **GM volume only** |  |  |  |  | 0.019 |
| GM volume | 0.991 | [0.984, 0.998] | -2.68 | 0.007 |  |
| **NAWM volume only** |  |  |  |  | 0.008 |
| NAWM volume | 0.998 | [0.993, 1.004] | -0.60 | 0.55 |  |
| **CSF volume only** |  |  |  |  | 0.043 |
| CSF volume | 1.012 | [1.007, 1.017] | 4.97 | <0.001 |  |
| **Brain-PAD, GM volume** |  |  |  |  | 0.042 |
| Brain-PAD  GM volume | 1.061  0.992 | [1.031, 1.092]  [0.986, 0.998] | 4.00  -2.52 | <0.001  0.01 |  |
| **Brain-PAD, CSF volume** |  |  |  |  | 0.050 |
| Brain-PAD +  CSF volume | 1.034  1.010 | [1.002, 1.067]  [1.004, 1.015] | 2.09  3.45 | 0.036  <0.001 |  |
| **GM volume, CSF volume** |  |  |  |  | 0.067 |
| GM volume +  CSF volume | 0.987  1.015 | [0.980, 0.993]  [1.010, 1.020] | -4.03  5.86 | <0.001  <0.001 |  |
| **Brain-PAD, GM volume, CSF volume** |  |  |  |  | 0.071 |
| Brain-PAD  GM volume  CSF volume | 1.026  0.987  1.013 | [0.994, 1.059]  [0.981, 0.994]  [1.008, 1.019] | 1.57  -3.78  4.55 | 0.12  <0.001  <0.001 |  |

Models were based on participants with complete neuroimaging data (N = 655, 73 deceased). Results are from Cox proportional hazard regression models of survival duration. All the above models included age at scan and sex as additional predictor variables.

## Supplementary Table 4. Brain-PAD and ageing biomarker mortality prediction model comparison

| Model | AUC | 95% Confidence Interval | Comparison vs. model 1 | Comparison vs. model 3 | Comparison vs. model 4 | Comparison vs. model 5 |
| --- | --- | --- | --- | --- | --- | --- |
| **1) Brain-PAD only** | 0.66 | 0.58, 0.73 | - | ↓ *P* <0.001 | ↓ *P* = 0.002 | ↑ *P* <0.001 |
| **3) Brain-PAD, DNAm-PAD, Telomere length** | 0.50 | 0.42, 0.58 | ↑ *P* <0.001 | - | No difference | ↑ *P* <0.001 |
| **4) Brain-PAD, DNAm-PAD** | 0.69 | 0.62, 0.76 | ↑ *P* = 0.002 | No difference | - | ↑ *P* <0.001 |
| **5) DNAm-PAD only** | 0.59 | 0.52, 0.66 | ↓ *P* <0.001 | ↓ *P* <0.001 | ↓ *P* <0.001 | - |

Model comparison based on same dataset with missing data excluded casewise (N = 620, 68 deceased). 95% confidence intervals were calculated using the DeLong method. Comparison were made using an analysis of deviance, based on the log likelihood of each model. Model number corresponds to Supplementary Table 1.

## Supplementary Table 5. Data sources for brain age training sample

| Cohort | N | Age  mean (SD) | Age  range | Sex  male/female | Repository details | Scanner  (Field strength) | Scan | Voxel dimensions |
| --- | --- | --- | --- | --- | --- | --- | --- | --- |
| ABIDE (Autism Brain Imaging Data Exchange) | 184 | 25.93 (6.66) | 18-48 | 161/23 | INDI | Various (all 3T) | MPRAGE | Various |
| Beijing Normal University | 179 | 21.25 (1.92) | 18-28 | 72/107 | INDI | Siemens (3T) | MPRAGE | 1.33x1.0x1.0 |
| Berlin School of Brain & Mind | 49 | 30.99 (7.08) | 20-60 | 24/25 | INDI | Siemens Tim Trio (3T) | MPRAGE | 1.0x1.0x1.0 |
| CADDementia | 12 | 62.33 (6.26) | 55-79 | 9/3 | http://caddementia.grand-challenge.org | GE Signa (3T) | 3D IR-FSPGR | 0.9x0.9x1.0 |
| Cleveland Clinic | 31 | 43.55 (11.14) | 24-60 | 11/20 | INDI | Siemens Tim Trio (3T) | MPRAGE | 2.0x1.0x1.2 |
| ICBM (International Consortium for Brain Mapping) | 322 | 24.84 (5.14) | 24-60 | 177/145 | LONI IDA | Siemens Magnetom (1.5T) | MPRAGE | 1.0x1.0x1.0 |
| IXI (Information eXtraction from Images) | 561 | 48.62 (16.49) | 20-86 | 250/311 | http://biomedic.doc.ic.ac.uk/brain-development | Philips Intera (3T); Philips Gyroscan Intera (1.5T); GE Signa (1.5T) | T1-FFE; MPRAGE | 0.9375x0.93751x1.2 |
| MCIC (MIND Clinical Imaging Consortium) | 93 | 32.49 (11.95) | 18-60 | 64/29 | COINS | Siemens Sonata/Trio (1.5/3T); GE Signa (1.5T) | MPRAGE; SPGR | 0.625x0.625x1.5 |
| MIRIAD (Minimal Interval Resonance Imaging in Alzheimer's Disease) | 23 | 69.66 (7.18) | 58-85 | 12/11 | https://www.ucl.ac.uk/drc/research/miriad-scan-database | GE Signa (1.5T) | 3D IR-FSPGR | 0.9375x0.93751x1.5 |
| NEO2012 (Adelstein, 2011) | 39 | 29.59 (8.38) | 20-49 | 18/21 | INDI | Siemens Allegra (3T) | MPRAGE | 1.0x1.0x1.0 |
| Nathan Kline Institute (NKI) / Rockland | 160 | 41.49 (18.08) | 18-85 | 96/64 | INDI | Siemens Tim Trio (3T) | MPRAGE | 1.0x1.0x1.0 |
| OASIS (Open Access Series of Imaging Studies) | 288 | 44.06 (23.04) | 18-90 | 106/188 | http://www.oasis-brains.org/ | Siemens Vision (1.5T)* | MPRAGE | 1.0x1.0x1.25 |
| WUSL (Power, 2012) | 24 | 23.04 (1.42) | 20-24 | 4/20 | INDI | Siemens Tim Trio (3T) | MPRAGE | 1.0x1.0x1.0 |
| TRAIN-39 | 36 | 22.67 (2.56) | 18-28 | 11/25 | INDI | Siemens Allegra (3T) | MPRAGE | 1.33x1.33x1.3 |
| Training set total | **2001** | **36.95 (18.12)** | **18-90** | **1016/985** | **-** | **-** | **-** | **-** |
| INDI = International Neuroimaging Data-sharing Initiative (<http://fcon_1000.projects.nitrc.org>)  COINS = Collaborative Informatics and Neuroimaging Suite ([http://coins.mrn.org](http://coins.mrn.org/))  LONI = Laboratory of Neuro Imaging Image & Data Archive (<https://ida.loni.usc.edu)>  ABIDE consortiums comprising data from various sites with different scanners/parameters  *OASIS scans were acquired four times and then averaged to increase signal-to-noise ratio. | | | | | | | | |
